# Differential modulation of cell morphology, migration, and Neuropilin-1 expression in cancer and non-cancer cell lines by substrate stiffness

**DOI:** 10.3389/fcell.2024.1352233

**Published:** 2024-06-05

**Authors:** Ana Monserrat Vela-Alcántara, Juan Santiago-García, Madeleine Barragán-Palacios, Aylin León-Chacón, Marilú Domínguez-Pantoja, Irene Barceinas-Dávila, Enrique Juárez-Aguilar, Elisa Tamariz

**Affiliations:** ^1^ Programa de Doctorado en Ciencias de la Salud, Instituto de Ciencias de la Salud, Universidad Veracruzana, Xalapa, Mexico; ^2^ Laboratorio de Cultivo Celular, Departamento de Biomedicina, Instituto de Ciencias de la Salud, Universidad Veracruzana, Xalapa, Mexico; ^3^ Laboratorio de Biología Molecular, Instituto de Investigaciones Biológicas, Universidad Veracruzana, Xalapa, Mexico; ^4^ Programa de Maestría en Ciencias de la Salud, Instituto de Ciencias de la Salud, Universidad Veracruzana, Xalapa, Mexico; ^5^ Facultad de Medicina, Universidad Veracruzana, Xalapa, Mexico

**Keywords:** substrate stiffness, cancer, neuropilin-1, mechanoresponse, migration

## Abstract

Physical changes in the tumor microenvironment, such as increased stiffness, regulate cancer hallmarks and play an essential role in gene expression, cell morphology, migration, and malignancy. However, the response of cancer cells to stiffness is not homogeneous and varies depending on the cell type and its mechanosensitivity. In this study, we investigated the differential responses of cervical (HeLa) and prostate (PC-3) cancer cell lines, as well as non-tumoral cell lines (HEK293 and HPrEC), to stiffness using polyacrylamide hydrogels mimicking normal and tumoral tissues. We analyzed cell morphology, migration, and the expression of neuropilin 1 (NRP1), a receptor involved in angiogenesis, cell migration, and extracellular matrix remodeling, known to be associated with cancer progression and poor prognosis. Our findings reveal that NRP1 expression increases on substrates mimicking the high stiffness characteristic of tumoral tissue in the non-tumoral cell lines HPrEC and HEK293. Conversely, in tumoral PC-3 cells, stiffness resembling normal prostate tissue induces an earlier and more sustained expression of NRP1. Furthermore, we observed that stiffness influences cell spreading, pseudopodia formation, and the mode of cell protrusion during migration. Soft substrates predominantly trigger bleb cell protrusion, while pseudopodia protrusions increase on substrates mimicking normal and tumor-like stiffnesses in HPrEC cells compared to PC-3 cells. Stiffer substrates also enhance the percentage of migratory cells, as well as their velocity and total displacement, in both non-tumoral and tumoral prostate cells. However, they only improve the persistence of migration in tumoral PC-3 cells. Moreover, we found that NRP1 co-localizes with actin, and its suppression impairs tumoral PC-3 spreading while decreasing pseudopodia protrusion mode. Our results suggest that the modulation of NRP1 expression by the stiffness can be a feedback loop to promote malignancy in non-tumoral and cancer cells, contingent upon the mechanosensitivity of the cells.

## 1 Introduction

Cancer stands as one of the leading causes of morbidity and mortality globally, with nearly 19 million new cases and 10 million deaths according to Globocan (2020). Prostate and cervical cancer rank as the fourth and seventh most common forms of cancer, respectively (Ferlay et al., 2020), imposing substantial costs on healthcare systems. Cancer is characterized by specific biological hallmarks crucial for the development, growth, and spread of tumor cells. Recent research has elucidated the significant influence of the biochemical and biophysical cues from the tumor microenvironment, particularly from the extracellular matrix (ECM), on these cancer hallmarks, underscoring its pivotal role in malignancy (Hanahan and Weinberg, 2011; Pickup et al., 2014a). The ECM, a non-cellular component, provides both biochemical and biomechanical support to cells, playing an indispensable role in morphogenesis, differentiation, and cellular homeostasis (Frantz et al., 2010). Moreover, the ECM plays a crucial role in tumor cell survival, invasion, migration, and ultimately, tumor metastasis (Mierke et al., 2008; Bissell and Hines, 2011; Pickup et al., 2014).

Several studies have highlighted that malignant tumors, such as prostate or breast cancer, exhibit higher stiffness compared to normal tissue, and even benign tumors (Phipps et al., 2005; Mouw et al., 2014). This increase in tumor stiffness has been associated with a more aggressive and metastatic cell phenotype (Pickup et al., 2014a; Mouw et al., 2014). The mechanical properties of the extracellular microenvironment influence tissue-specific gene expression responsible for maintaining tissue integrity (Alcaraz et al., 2008), thereby modulating biological processes like proliferation, migration, and metastasis in breast, hepatic, and other cancer cells (Tilghman et al., 2010; Schrader et al., 2011; Feng et al., 2013; Ansardamavandi et al., 2018). Furthermore, an increase in ECM stiffness triggers changes in phenotypic characteristics (Tilghman et al., 2010; 2012), promotes epithelial-to-mesenchymal transition (EMT), and has been implicated in regulating the expression of stemness-associated genes (Schrader et al., 2011; Hui et al., 2017; Piao et al., 2017; Rice et al., 2017).

Although the stiffness of the microenvironment influences tumor cell biology, responses could be differential and heterogeneous among tumor cells with different malignancies, as demonstrated by studies on patient-derived glioblastoma cell lines (Grundy et al., 2016). It has been observed that stiffer substrates promote the proliferation of various cancer cell lines including PC-3, DU145, SiHa, HeLa, MDA-MB-231, and A549, while higher stiffness has no significant impact on proliferation in LNCaP, C4-2B, SW620 and mPanc236 cell lines (Tilghman et al., 2012; Pasqualato et al., 2013; Liu et al., 2020; Jin et al., 2022). Furthermore, cell-substrate interaction, such as the traction forces exerted by the cells, depends on the cell characteristics such as the metastatic potential. For instance, breast, prostate, and pulmonary metastatic cells (MDA-MB-231, PC-3, and A549) increase their traction forces with increasing substrate stiffness, unlike their normal epithelial counterparts (MCF-10A, HPrEC, and BEAS2B cells). Additionally, traction forces vary with metastatic potential, being higher in high metastatic potential cell lines like MDA-MB-231compared to low metastatic potential cell lines like MDA-MB-468, and even lower in benign cell line MCF-10A (Kraning-Rush et al., 2012; Kristal-Muscal et al., 2013).

Similarly, studies have shown that the migration process known as durotaxis, where cells move towards rigid environments, can be counteracted in certain cell types. For instance, the glioblastoma cell line U-251MG, isolated from the brain tissue with low stiffness, exhibits this behavior (Tabdanov et al., 2018). Even MDA-MB-468 breast cancer cells, which typically undergo durotaxis, can alter their cytoskeleton molecular machinery when cultured on soft substrates, favoring migration characterized by a ligand-dependent dendritic phenotype over myosin/actin contraction and focal adhesion-dependent migration observed on stiffer substrates (Isomursu et al., 2022). These findings highlight the diverse responses of cells to substrate stiffness and their ability to adapt molecular mechanisms to support specific processes such as proliferation or migration. Moreover, the heterogeneity of the responses may be linked to the cell’s origin. Thus, the study of the effects exerted by the biomechanics of the tumor microenvironment on the cells is complex and requires further investigation.

Among the proteins implicated in cancer progression, the neuropilin-1 receptor (NRP1) has been linked to facilitating tumor proliferation, invasion, migration, and EMT (Chu et al., 2014; Zhang et al., 2016a; Matkar et al., 2016; Wu et al., 2020). NRP1 is a transmembrane glycoprotein expressed in both vertebrate embryonic and adult tissues (Zhang et al., 2016). During embryonic development, NRP1 plays an important role in neuronal cell migration by binding to class 3 semaphorin ligands and in angiogenesis through its interaction with VEGF_165_ (Kawakami et al., 1996; Kawasaki et al., 1999; Niland and Eble, 2019). In adult tissues, it is predominantly expressed in endothelial artery cells (Kawasaki et al., 1999; Zeng et al., 2014). Several studies have reported a significant increase in NRP1 expression during tumorigenesis (Kawasaki et al., 1999; Zeng et al., 2014), with this overexpression correlating directly with decreased patient survival (Bachelder et al., 2001; Yaqoob et al., 2012). Consequently, it has garnered attention as a potential target for the development of new chemotherapeutic drugs (Hong et al., 2007; Pan et al., 2007; Wild et al., 2012; Graziani and Lacal, 2015; Li et al., 2016; Meyer et al., 2016).

NRP1 expression has been associated with the metastatic potential of prostate cancer cells; more aggressive cell lines such as DU145 or PC-3 exhibit higher expression levels compared to less aggressive cell lines and benign hyperplasia cells (Tse et al., 2017). Furthermore, it has been proposed that NRP1 binding to fibronectin (FN), through activation of α5β1 integrin, promotes ECM remodeling, rendering it insoluble and stiffer, thereby triggering a fibrotic reaction that favors tumor growth (Yaqoob et al., 2012). Interestingly, in previous research, we observed that substrate stiffness regulates NRP1 expression in embryonic neurons, with an increase in NRP1 expression when cultured in stiffer substrates (Vela-Alcantara et al., 2022). In the present study, we assess the differential response of tumoral and non-tumoral cells to substrate stiffness, analyzing whether stiffness modulates NRP1 expression and therefore related to the malignancy and metastatic potential of the cells. Our findings reveal, for the first time, a differential regulation of NRP1 driven by substrate stiffness in non-tumoral and cancer cells, alongside varying impacts of stiffness on cell morphology, actin remodeling, and cell migration.

## 2 Materials and methods

### 2.1 Polyacrylamide hydrogels

Polyacrylamide (PAA) hydrogels were prepared with stiffness ranging from 0.15, 3.43, and 80 kPa, following established protocols (Pelham and Wang, 1997; Cozzolino et al., 2016). Briefly, 12- or 20-mm round coverslips were wiped with 0.1 N NaOH and then treated with (3-aminopropyl) triethoxysilane (APTES; Sigma-Aldrich, St. Louis, MO). After washing the coverslips three times, the glass surface was treated with 0.5% glutaraldehyde for 30 min and allowed to dry at room temperature. To create PAA hydrogels with different stiffness, mixtures of 40% acrylamide/2% bisacrylamide (Sigma-Aldrich, St. Louis, MO) were prepared with sterile distilled water to a final volume of 1 mL. The solution was degassed for 8–15 min before polymerization was initiated by adding freshly prepared ammonium persulfate (APS; 1% w/v solution; Bio-Rad Laboratories, Inc., Hercules, CA) and N, N, N′, N′-tetramethylenediamine (TEMED; 0.1% w/v solution; Sigma-Aldrich, St. Louis, MO). After a brief agitation, PAA solution was added onto 12 mm × 15 mm coverslips previously coated with dichlorodimethylsilane (DCDMS; Sigma-Aldrich, St. Louis, MO). Then the 12 or 20 mm functionalized round coverslips were placed over each hydrogel droplet of PAA solution and allowed to polymerize for 30 min. Once polymerization was complete, round coverslips with the PAA hydrogel were carefully removed and placed in 24- or 12-well culture plates, washed three times with 50 mM HEPES (J.T. Baker, Thermo Fisher Scientific Inc., Phillipsburg, NJ) at pH 8.5. PAA hydrogels were photoactivated using a 1 mM solution of sulfosuccinimidyl 6-(4′-azido-2′-nitrophenylamino)hexanoate (sulfo-SANPAH; Sigma-Aldrich, St. Louis, MO) in 50 mM HEPES under UV light for 8 min, repeated twice, and then washed three times with the HEPES solution. Finally, all PAA hydrogels were coated with poly-L-lysine (PLL 500 μg/mL; Sigma-Aldrich, St. Louis, MO) for 30 min, washed three times with 1× Phosphate Buffered Saline (PBS) at pH 7.4, and incubated with RPMI 1640 growth culture media for 1 h before cell seeding.

### 2.2 Cell lines

All cell lines used in this study were obtained commercially from ATCC, including tumoral human cell lines: epithelioid cervix carcinoma cells (HeLa, ATCC CRL-CCL-2) and a metastatic prostate cancer cell line (PC-3, ATCC CRL-1435), as well as two non-tumoral human cell lines: a normal human primary prostate epithelial cell line (HPrEC, ATCC PCS-440-010) and human embryonic kidney 293 cells (HEK293, ATCC CRL-157340). Cell lines were authenticated by the National Institute of Genomic Medicine (INMEGEN, Mexico) before conducting the assays.

HEK293 is a transformed cell line derived from isolated human embryo kidney cells and is considered non-tumorigenic within 52 passages; tumors are induced in nude mice only with high passage cells, as previously reported (Shen et al., 2008). HPrEC cells are derived from normal primary prostate epithelial cells, exhibiting normal epithelial morphology, and a limited lifespan in culture (Gil et al., 2005). HeLa cells originate from cervical carcinoma and are highly tumorigenic, widely used as a model for tumor formation (Kusakawa et al., 2015). PC-3 cells, derived from bone metastasis of grade IV prostatic adenocarcinoma, lack androgen receptor and prostate-specific antigen and are considered highly metastatic and tumorigenic (Kaighn et al., 1979).

Cells were cultured in RPMI-1640 medium (Sigma-Aldrich, St. Louis, MO), or prostate epithelial cell basal medium (Prostate Epithelial cell Growth kit, ATCC PCS-440-040) for HPrEC, supplemented with 8% fetal bovine serum (FBS; Biowest, Nuaillé, France) and 1% penicillin/streptomycin (Gibco, Life Technologies Corporation, Grand Island, NY), and maintained in a 37°C/5% CO_2_ atmosphere. Cells from passages 2-10 were seeded on the PAA hydrogels at a density of 1 × 10^4^ cells per cm^2^ and incubated at 37°C/5% CO_2_ for 24 or 48 h.

### 2.3 Morphometric analyses

The cells from all 4 cell lines were fixed with 4% paraformaldehyde (PFA, Sigma-Aldrich, St. Louis, MO) after being seeded on the 12 mm PAA hydrogels for 24 h. Micrographs were captured using a Nikon Eclipse TS100 phase contrast microscope (Nikon Instruments Inc, Japan) equipped with a CCD camera, using a 20x lens. The Fiji software (ImageJ2, Wane Rasband, U. S. National Institutes of Health, Bethesda, MD) was used for image analysis (Schindelin et al., 2012).

Morphological parameters quantified included cell area, aspect ratio (ratio of the major axis to the minor axis of the ellipse for each cell), circularity (expressed as 4π times the ratio of the area to the perimeter, where a value close to 1 indicates a perfect circle and close to 0 indicates greater elongation of the cell), and solidity (calculated by dividing the cell area by its convex area, assuming the inner angles are less than 180°, convex and that it contains the original region; a more branched cell results in a larger convex area and a lower solidity value) (Zanier et al., 2015). At least 12 images per coverslip were captured. Three independent experiments were performed, each with triplicates per condition.

### 2.4 RNA isolation, cDNA synthesis, and Real-time PCR (qRT-PCR)

Total RNA extraction from cultured cells on PAA hydrogels at different time points was conducted following the TRIsure RNA isolation protocol (Bioline Reagents Ltd., London, United Kinngdom). RNA concentration and purity were determined using a NanoDrop ND-1000 (Thermo Fisher Scientific, Waltham, Massachusetts, United States). cDNA was synthesized by random priming from 500 ng of total RNA using M-MLV Reverse Transcriptase (Invitrogen, ThermoFisher Scientific, Waltham, Massachusetts, United States), according to the manufacturer’s instructions.

Real-time PCR reactions were performed in triplicate with 1 µL of cDNA, 5.5 µL of 2 × SensiFast SYBR qPCR mix (Green Bioline), 5 pmol of primers, and water up to 10 µL. The sequences for each pair of primers used were: NRP1 (forward 5′-AGG​ACA​GAG​ACT​GCA​AGT​ATG​AC-3’; reverse 5′-AAC​ATT​CAG​GAC​CTC​TCT​TGA-3′) and the values of mRNA expression were normalized to that of 18S rRNA (forward 5′-GTA​ACC​CGT​TGA​ACC​CCA​TT-3′; reverse 5′-CCA​TCC​AAT​CGG​TAG​TAG​CG-3′) or YWHAZ, as it has been reported as a reliable reference gene for cancer cells (Chua et al., 2011), (forward 5′-TTG​AGC​AGA​AGA​CGG​AAG​GT-3′; reverse 5′-GAA​GCA​TTG​GGG​ATC​AAG​AA-3′). The cycling conditions for qPCR were: 2 min at 95°C, 40 cycles of 15 s at 95°C, and 1 min at 60°C in a 7500 Real-Time PCR System (Applied Biosystems, Foster City, CA). PCR efficiencies were calculated using the LinReg program (Ruijter et al., 2009), and relative gene expression was calculated with the Pfaffl equation (Pfaffl, 2001). Data were normalized according to the expression in cells cultured over the softest stiffness condition (0.15 kPa) at 12 or 24 h. Three independent experiments with triplicates were performed.

### 2.5 Flow cytometry

Cells cultured for 24 and 48 h on PAA hydrogels with different stiffness (0.15 kPa, 3.43 kPa, and 80 kPa) were immunostained with the anti-NRP1 antibody (APC anti-human CD304; Neuropilin-1, BioLegend, catalog 354505 or PE anti-Nrp1; R&D Systems, catalog FAB566P). After culture time, each coverslip was transferred to a clean sterile well, washed twice with 1 × PBS, and treated with trypsin/EDTA 0.25% solution (Gibco, Life Technologies Corporation, Grand Island, NY) or trypsin/EDTA for primary HPrEC cells until the cells detached from PAA hydrogel. Trypsin was then inactivated by adding an equal volume of RPMI medium with 8% FBS, or 1% trypsin neutralizing solution for HPrEC cells. The cell suspension was centrifuged for 5 min at 300 xg, washed with 1x PBS at 37°C, and the supernatant was discarded. Cells were stained for 45 min using the anti-NRP1 antibody. After the incubation, the cells were washed with 1x PBS with 1% albumin (1% PBA), centrifuged for 5 min at 300 xg and the supernatant was discarded. Cells were fixed with 4% formaldehyde (FA; ThermoFisher Scientific, catalog 28906). Flow cytometry acquisitions were performed using a BD LRS Fortessa flow cytometer (Becton-Dickinson Bioscience, San Jose, CA) and analyzed with the FlowJo software v10.6.2 (FlowJo, TreesStar, Ashland, OR). Three independent experiments were performed for each condition.

### 2.6 Migration assay

Cell migration was assessed using time-lapse microscopy in cells cultured for 48 h in PAA hydrogels (0.15 kPa, 3.43 kPa, and 80 kPa) on 20 mm diameter coverslips. The coverslips with the cells were transferred to an incubation chamber, and growth media with HEPES (final concentration of 0.01 M) was added. The chamber was then mounted on a stagetop incubator, and phase-contrast microscope images were captured at 20-s intervals, with a total recording time of 2 h 30 min. Image analysis was performed using the Fiji software (ImageJ2, Wane Rasband, U. S. National Institutes of Health, Bethesda, MD).To distinguish between migratory and non-migratory cells, a box encompassing the nucleus of each cell was delineated. Cells that displaced their nucleus at least one-third outside the box during the recording period were considered migratory (Grundy et al., 2016). Total distance and average displacement speed were quantified for each cell.

Manual tracking of the cell movement of each migratory cell was carried out using the Trackmate (Manual Tracking) plug-in of the Fiji software (Tinevez et al., 2017; Ershov et al., 2021). To visualize the cell displacement trajectories, the trajectory of each cell was transposed to a common origin using the Excel macro Diper Plot_At_Origin.txt (Gorelik and Gautreau, 2014), and plots were generated based on the x, and y coordinates of the trajectory data. Furthermore, using the Excel macro MSD.txt of the Diper program (Gorelik and Gautreau, 2014), the area explored by each cell was quantified by plotting the mean square displacement (MSD) against half the recording time (75 min). From the MSD, the slope α of the log-log curve of the MSD data was calculated for the cells in each stiffness condition. This value was used to determine the persistence of cell movement, where α = 1 represents random movement and α = 2 represents directed motion. Three independent experiments were performed for each condition.

### 2.7 Immunostaining

PC-3 cells cultured for 24 h on PAA hydrogels with different stiffness (0.15 kPa, 3.43 kPa, and 80 kPa), or silenced cells cultured for 48 h, were fixed with methanol free 4% formaldehyde (Pierce™, ThermoFisher Scientific, catalog 28906), blocked with 5% horse serum in 1x PBS, and immunostained overnight with 1:500 dilution of anti-NRP1 antibody (anti-human Neuropilin 1, R&D systems, catalog AF3870) in 5% horse serum in 1x PBS. After several washes with 1x PBS, cells were incubated with a 1:800 dilution of an anti-goat IgG coupled to Alexa Fluor 488 (Invitrogen, catalog A11055), washed again in 1x PBS, and incubated with phalloidin conjugated to Alexa Fluor 546 (Invitrogen, catalog A22283). Cell cultures were washed and mounted on coverslips with a drop of 90% glycerol containing Hoechst 33342 (Invitrogen, catalog H3570). Immunostained cells were observed using confocal microscopy (Leica TCS SP8 AOBS or Leica TSP8), with images acquired using a ×63 oil immersion objective. Z-stacks of 1 µm were obtained, and maximum projection images were generated using Fiji ImageJ software.

### 2.8 siRNA knockdown

PC-3 cells cultured on 12-well plates were transfected with either 20 or 30 pmol of Silencer^®^ Select Validated siRNA-NRP1 (Invitrogen) using Lipofectamine RNAiMax reagent (Invitrogen, catalog 13778075). Alternatively, cells were double transfected with a non-relevant siRNA coupled to Cy3 (Invitrogen, catalog AM4620) and siRNA-NRP1. After 24 h of transfection, cells were detached and seeded onto PAA hydrogels. They were incubated for 48 h and transferred to the incubation chamber for time-lapse migration assays, or fixed for immunostaining.

### 2.9 Statistical analysis

The data were assessed for normality using either the Kolmogorov-Smirnov or Shapiro-Wilk test. Normally distributed data were analyzed using the one-way ANOVA test, followed by Tukey’s multiple comparisons test or Student’s t-test. For data that were not normally distributed, Kruskal-Wallis’s test was used, followed by Dunn’s multiple comparisons test with the Bonferroni correction (Dunn Bonferroni’s test). Differences with a value of *p* < 0.05 were considered significant. All analyses were performed using IBM SPSS Statistics 20.0 (IBM Corp., Armonk, NY), and graphs were generated using GraphPad Prism 9.0.2 (GraphPad Software, La Jolla, CA).

## 3 Results

### 3.1 Stiffer substrates increase cell spreading and ramification in tumoral and non-tumoral cells but within different stiffness ranges

**FIGURE 1:**
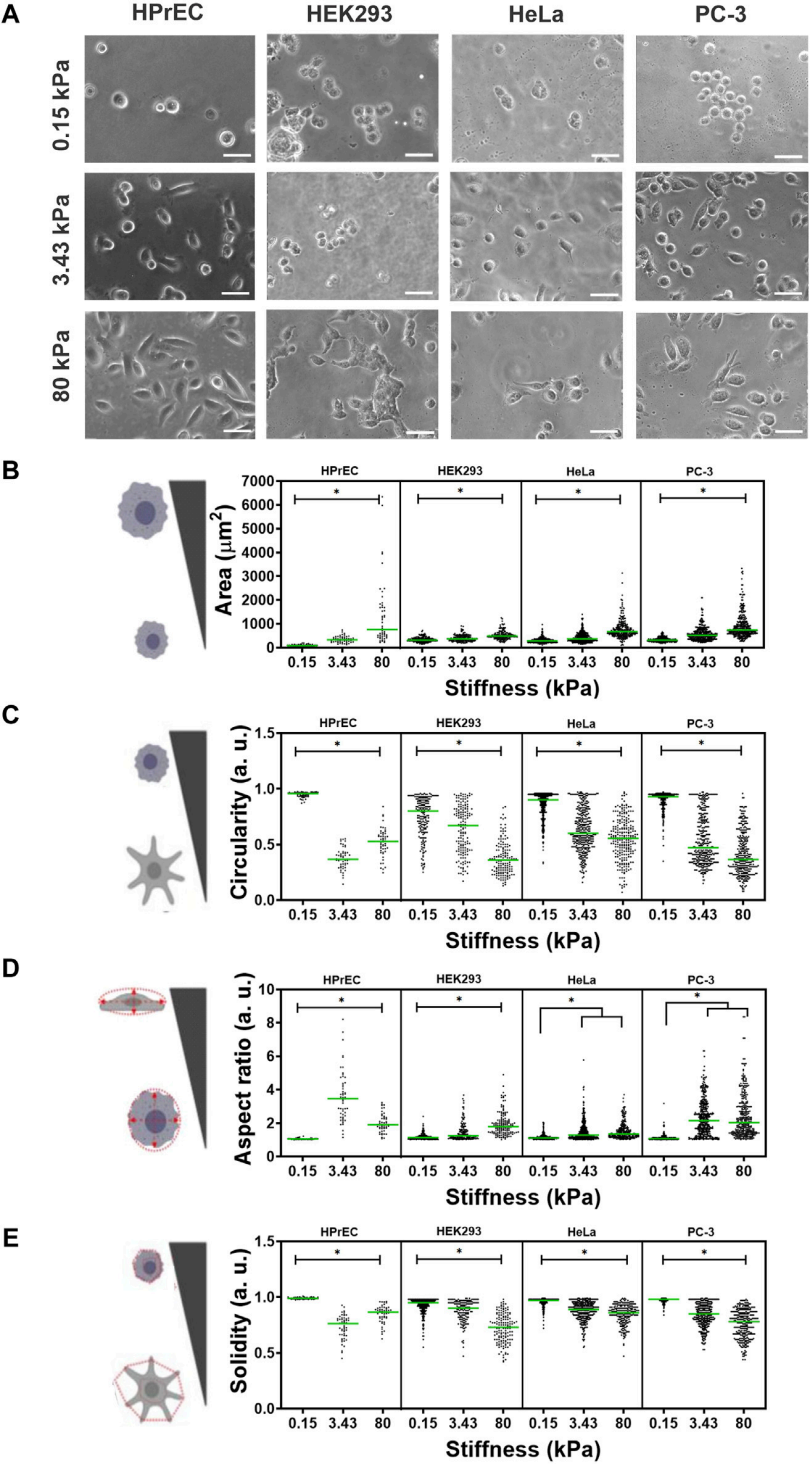
Effect of substrate stiffness on cell morphology of HPrEC, HEK293, HeLa, and PC-3 cell lines cultured over PAA hydrogels with different stiffness. **(A)** Representative phase contrasts images of cells after 24 h of incubation at the indicated stiffness. Scatter plots with median values (green bar) depict the quantification of morphological parameters **(B)** area, **(C)** circularity, **(D)** aspect ratio, and **(E)** cell solidity on PAA hydrogels of different stiffness. Data obtained from three independent experiments conducted in triplicate were analyzed with Kruskal-Wallis followed by Dunn-Bonferroni’s multiple comparisons tests, *p < 0.05. HPrEC: 0.15 kPa, *n* = 60; 3.43 kPa, *n* = 54; 80 kPa, *n* = 70. HEK293: 0.15 kPa, *n* = 201; 3.43 kPa, *n* = 159; 80 kPa, *n* = 148. HeLa: 0.15 kPa, *n* = 335; 3.43 kPa, *n* = 375; 80 kPa, *n* = 246. PC-3: 0.15 kPa, *n* = 342; 3.43 kPa, *n* = 387; 80 kPa, *n* = 332. Scale bar in **(A)** = 50 µm.

To compare the influence of stiffness on cell morphology, we examined non-tumoral cell lines HPrEC and HEK293, as well as tumoral HeLa and PC-3 cells, cultured on PAA hydrogels with varying stiffness, a low stiffness, non-related to the tissue of origin of the cell lines (0.15 kPa), an intermediate stiffness (3.43 kPa) akin to normal prostatic or cervical tissue (Jiang et al., 2014; Piao et al., 2017), and a higher stiffness (80 kPa), associated with prostate and cervical tumors (Porsch et al., 2015; Cui et al., 2017; Rouvière et al., 2017). Phase contrast micrographs revealed that substrate stiffness significantly influenced cell morphology across all cell lines. Specifically, all the cells appeared more rounded and smaller on softer substrates (0.15 kPa). Notably, HEK293 and HeLa cells remain mainly rounded even on the intermediate stiffness (3.43 kPa), while some HPrEC and PC-3 cells exhibited a less rounded and more extended morphology at this stiffness. At the highest stiffness (80 kPa), all cell lines displayed a similar morphology (Figure 1A).

Quantitative analysis of four morphological parameters shows that cell area increased as PAA hydrogel stiffness increased in all cell lines (Figure 1B). Moreover, cell circularity decreased with increasing substrate stiffness, and cells were less rounded as the substrate stiffness increased, being more evident in HPrEC and PC-3 cells (Figure 1C). The aspect ratio, a measure of cell elongation, increased with stiffness, albeit without significant differences observed between intermediate and higher stiffness in HeLa and PC-3 cells. Noteworthy was the higher elongation of HPrEC cells at the intermediate stiffness (3.43 kPa) compared to other cell lines (Figure 1D). Additionally, cell solidity, a descriptor of cell deformability potentially relevant to metastatic spread (Pasqualato et al., 2013), decreased (indicating increased deformability), with increasing stiffness in HEK293, HeLa, and PC-3 cells, while only HPrEC cells showed decreased solidity at the intermediate stiffness (3.43 kPa) compared to the stiffer substrate (Figure 1E).

These findings underscore the role of substrate stiffness in modulating the morphology of all the assayed cell lines, indicating that all were mechanoresponsive; however, there are discernible differences in sensitivity or response to stiffness among them, with HPrEC and PC-3 cells exhibiting more pronounced morphological changes at the intermediate and higher stiffness substrates (3.43 kPa and 80 kPa). Particularly notable is the elongated and branched morphology observed in HPrEC cells at the intermediate stiffness (3.43 kPa), in contrast to the other cell lines.

### 3.2 NRP1 expression increases on tumor-like stiffness in non-tumoral HPrEC and HEK293 cells and on normal tissue-like stiffness in tumoral PC-3 cells

**FIGURE 2:**
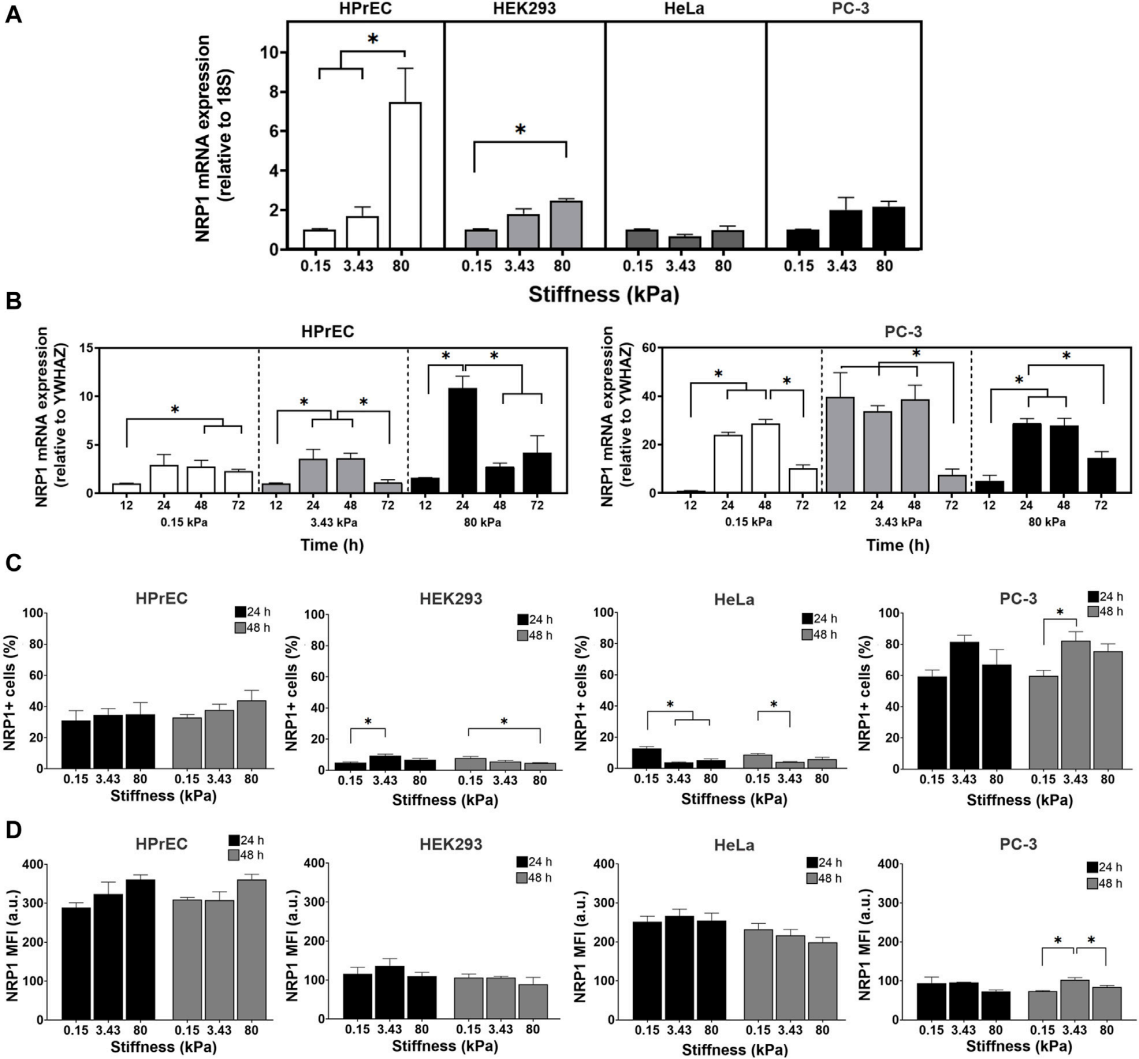
Regulation of NRP1 expression in cells cultured on substrates with different stiffness. **(A)** Relative expression of NRP1 transcript in HPrEC, HEK293, HeLa, and PC-3 cells cultured for 24 h on PAA hydrogels of the indicated stiffness. **(B)** Relative expression of NRP1 transcript in HPrEC and PC-3 cells cultured for 12–72 h on PAA hydrogels of the indicated stiffness, the expression in cells cultured over 0.15 kPa at 12 h was considered as 1. **(C)** Percentage of NRP1 expressing HPrEC, HEK293, HeLa, and PC-3 cells cultured on the PAA hydrogels for 24 and 48 h **(D)** NRP1 mean fluorescent intensity (MFI) of HPrEC, HEK293, HeLa, and PC-3 cells cultured for 24 and 48 h on PAA hydrogels measured by flow cytometry. *p < 0.05, **(A)** Kruskal-Wallis followed by Dunn-Bonferroni’s multiple comparisons test (HPrEC, HEK293, and PC-3) and One-way ANOVA with Tukey’s multiple comparisons test (HeLa); triplicates from three independent experiments, and **(B)** Kruskal-Wallis followed by Dunn-Bonferroni’s multiple comparisons tests (HPrEC 12, 24, 72 h and PC-3 12, 48 h) and One-way ANOVA with Tukey’s multiple comparisons test (HPrEC 48 h and PC-3 24, 72 h); triplicates from two independent experiments. **(C)** One-way ANOVA with Tukey’s multiple comparisons test from three independent experiments. MFI, mean fluorescent intensity.

To analyze whether substrate stiffness modulates the expression of NRP1, a protein associated with cancer progression, we analyzed NRP1 mRNA expression in four different cell lines following 24 h of culture on PAA hydrogels. Our findings revealed distinct patterns of NRP1 mRNA expression among the cell lines. Specifically, non-tumoral HPrEC and HEK293 cells exhibited a significant increase in mRNA expression as stiffness increased, whereas this difference was not observed in tumoral PC-3 and HeLa cells. Notably, HPrEC cells, a non-tumoral prostate epithelial cell line, demonstrated a remarkable increase in NRP1 mRNA expression when cultured on substrates mimicking tumor-like stiffness (80 kPa) (Figure 2A).

To further explore the impact of substrate stiffness on NRP1 mRNA expression we compared the expression of the non-tumoral HPrEC and tumoral PC-3 cells at different times of incubation. We observed variations in the peak expression of NRP1 mRNA in HPrEC and PC-3 cells, with distinct responses to both time and substrate stiffness. In PC-3 cells, the peak of expression occurred at the 3.43 kPa stiffness after 12 h of incubation, remaining stable until 48 h. Conversely, HPrEC cells exhibited a significant peak in expression at 80 kPa tumor-like stiffness substrate after 24 h of incubation (Figure 2B). These results confirm the impact of stiffness on NRP1 expression and highlight differential mRNA induction dynamics between non-tumoral and tumoral cells, suggesting that non-transformed cells require stiffer conditions and longer incubation times to increase NRP1 mRNA expression. In contrast, PC-3 tumoral cells, demonstrate increased NRP1 expression at stiffness akin to normal prostate tissue, and with shorter incubation periods.

Flow cytometry analysis of NRP1 protein expression revealed differences in the percentage of NRP1-expressing cells among the different cell lines. HEK293 and HeLa cells exhibited less than 20% NRP1-positive cells, with even lower percentages on stiffer gels, while HPrEC and PC-3 showed higher percentages, exceeding 20% and 50%, respectively, on softer gels, and about 40% and 80% on stiffer substrates (Figure 2C). Similar to mRNA findings, substrate stiffness exerts differential effects on NRP1 protein expression across cell lines. As stiffness increases, HEK293 cells at 24 h and PC-3 cells after 48 h of incubation displayed a higher percentage of positive cells. Conversely, NRP1 expression decreased with increasing stiffness in HEK293 cells at 48 h, as well as in HeLa cells at both 24 and 48 h (Figure 2C). Although HPrEC cells exhibited no changes in NRP1-positive cells across when cultured on the different substrate stiffnesses; however, a tendency toward increased expression on stiffer substrates was observed (Figure 2C). Mean fluorescent intensity (MFI) remains unchanged in HPrEC, HEK293, and HeLa cells under all conditions, suggesting that stiffness does not regulate the amount of protein at the cell membrane in these cell lines (Figure 2D). In contrast, in PC-3 cells, NRP1 MFI increased on the substrate with intermediate stiffness (3.43 kPa) compared to softer and stiffer substrates, indicating that this stiffness favored NRP1 protein expression in these cells.

### 3.3 PC-3 tumoral cells show predominant bleb protrusions on softer and normal tissue-like substrates and change to elongated pseudopodia protrusions on stiffer tumor-like substrates

Cell migration is a fundamental aspect related to tumor progression and metastasis. Thus, we examined whether substrate stiffness has differential effects on cell migration in both non-tumoral and tumoral cells. Specifically, we investigated the impact of substrate stiffness on the migration of HPrEC and PC-3 cells, given their sensitivity to stiffness, using time-lapse microscopy 48 h post culture on soft (0.15 kPa), intermediate (3.43 kPa), and stiff (80 kPa) PAA hydrogels.

**FIGURE 3:**
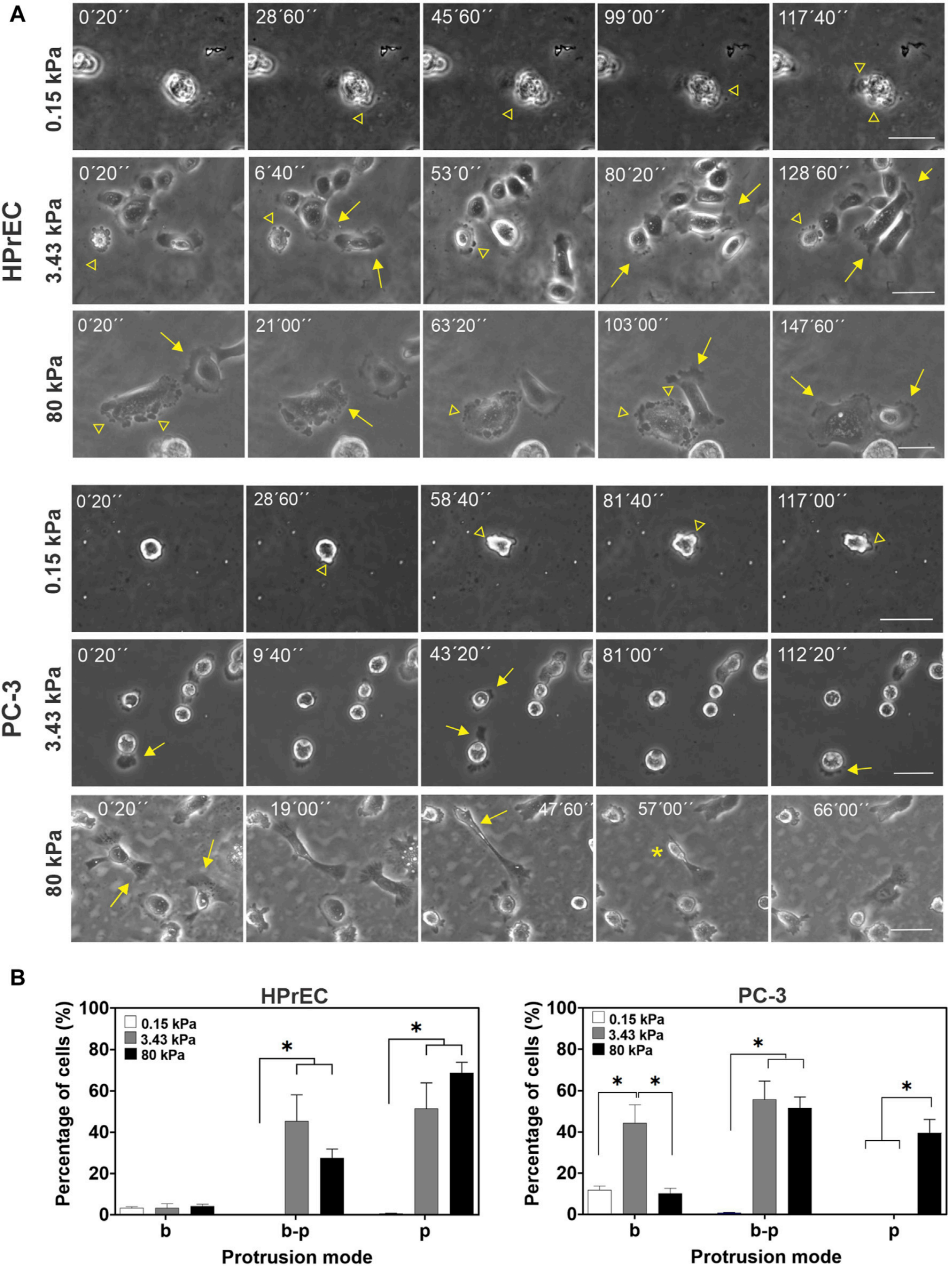
Modulation of cell protrusion by substrate stiffness. **(A)** Representative images from the time-lapse experiments of HPrEC and PC-3 cell lines cultured on different substrate stiffness. Arrowheads point to bleb protrusions, arrows point to pseudopodia protrusions, and an asterisk indicates a fast-retracting cell after forming an elongated pseudopodium. **(B)** Percentage of cells with only blebs (b), bleb-pseudopodia (b–p), or only pseudopodia (p) protrusions identified on time-lapse experiments of PC-3 and HPrEC cells on different substrate stiffness. One-way ANOVA with Tukey’s multiple comparisons test. Bars represent the mean ± SEM, *p < 0.05. HPrEC: 0.15 kPa, *n* = 156; 3.43 kPa, *n* = 156; 80 kPa, *n* = 165 cells. PC-3: 0.15 kPa, *n* = 151; 3.43 kPa, *n* = 264; 80 kPa, *n* = 261 cells from duplicates from three independent experiments. Scale bar = 50 μm.

Our first observation revealed different cell protrusion modes at the different substrate stiffnesses; cell membranes exhibited blebs, pseudopodia, or transitions between blebs and pseudopodia. In the softest PAA hydrogels, blebs were the predominant membrane protrusions in both HPrEC and PC-3 cells, with other protrusion modes emerging as the substrate stiffened (Figure 3A; Supplementary Videos SV1-S6). HPrEC cells show alternating blebs and lamellipodia, with a minority of cells maintaining solely blebs (Figure 3A). PC-3 cells exhibit blebs at 3.4 kPa, but an increasing number of cells displayed bleb-pseudopodia combinations; in some instances, small lamellipodia or filopodia were formed. Notably, at 80 kPa, certain PC-3 cells adopted an elongated morphology with small and active pseudopodia that abruptly retracted and impulse forward cell displacement (Figure 3A; Supplementary Video SV6). Quantitative analysis of the percentage of cells exhibiting each protrusion mode confirmed that PAA hydrogel stiffness influences protrusive activity. The proportion of PC-3 cells displaying only pseudopodia increases at 80 kPa, while decreasing the number of blebbing cells, more prevalent at 3.43 kPa. Conversely, HPrEC cells showed no significant changes in the percentage of protrusions on substrates with stiffnesses of 3.43 and 80 kP (Figure 3B).

### 3.4 Stiffness increases cell migration in non-tumoral and tumoral HPrEC and PC-3 cells but it only increases the persistence of migration in PC-3

**FIGURE 4:**
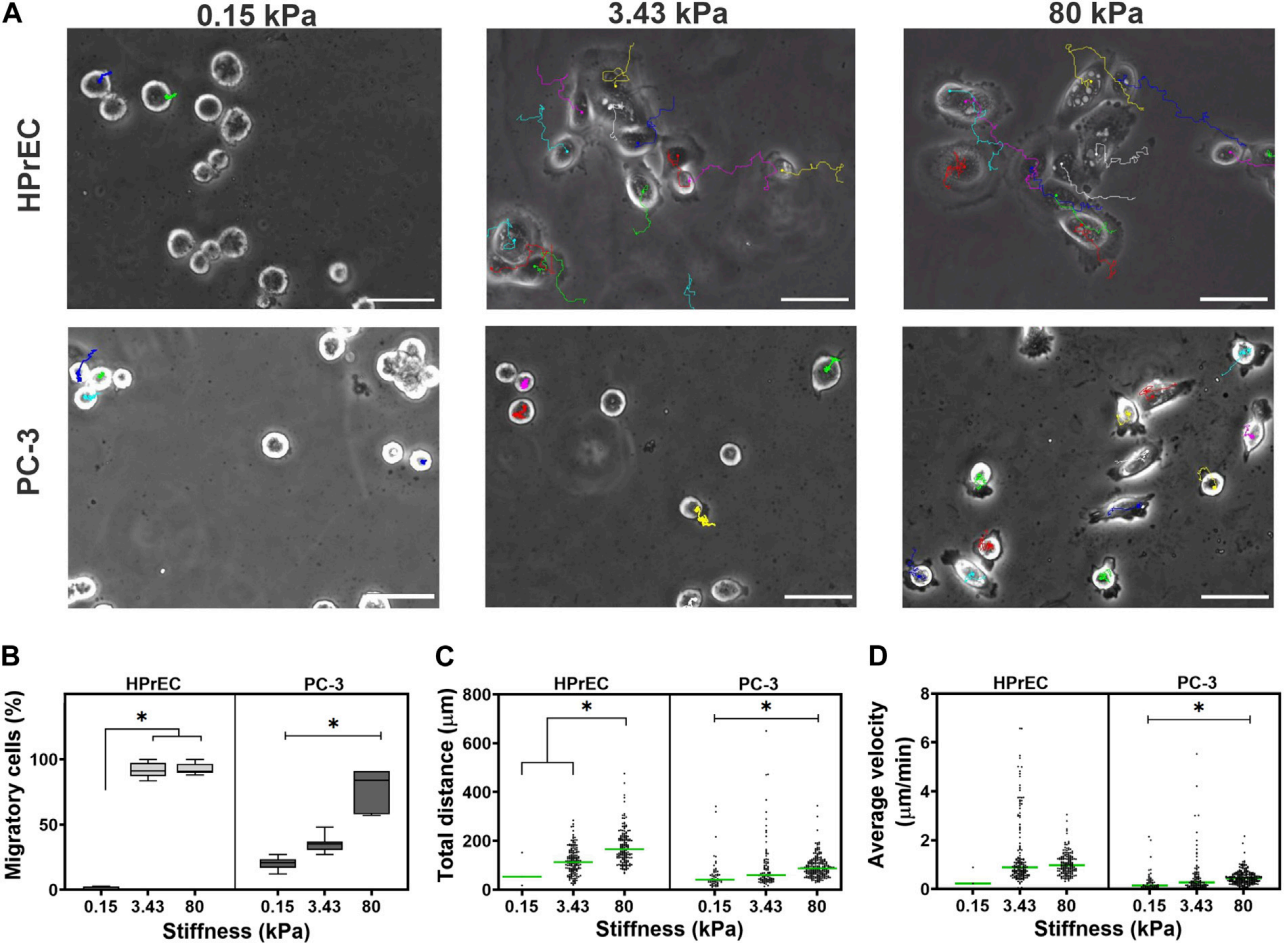
Modulation of migration in non-tumoral and tumoral cells by substrate stiffness. **(A)** Representative images from the time-lapse experiments of cell lines cultured on different substrate stiffness. The color lines in the images represent the displacement of each cell in time. **(B)** Box plots showing the percentage of migratory HPrEC and PC-3 cells cultured for 48 h on PAA hydrogels. Scatter plots showing the total distance traveled **(C)** and the average velocity of displacement **(D)** of HPrEC and PC-3 cells on the PAA hydrogels, green bars indicate the median. Data in **(B)** were analyzed by one-way ANOVA with Tukey’s multiple comparisons test, whereas **(C)** and **(D)** with Kruskal-Wallis with Dunn-Bonferroni multiple comparisons test; *p < 0.05. HPrEC: 0.15 kPa, *n* = 3; 3.43 kPa, *n* = 157; 80 kPa, *n* = 160. PC-3: 0.15 kPa, *n* = 58; 3.43 kPa, *n* = 93; 80 kPa, *n* = 222, from duplicates from three independent experiments. Scale bar = 50 μm.

Analysis of cell migration revealed that HPrEC cells exhibit high migratory behavior, with over 90% of cells migrating on both, intermediate and stiff substrates, but not on the softest gels, where only about 2% of the cells migrated. Similarly, PC-3 cell migration was influenced by substrate stiffness; a significantly higher percentage of migrating cells (78%) was observed on stiffer substrates compared to softer ones, where the percentages were 20% and 35%, respectively (Figure 4B). In migratory PC-3 cells, both total displacement distance and average velocity increased with the stiffness of PAA hydrogels, indicating that cells traveled further and at higher average velocity on stiffer substrates (Figures 4C,D). In contrast, HPrEC migratory cells traveled greater distances when cultured on 80 kPa stiffer substrate compared to cells cultured on 3.43 kPa substrate; however, there was no significant difference in average cell velocity among the substrate stiffnesses, although there was a tendency for increased velocity with increasing stiffness (Figures 4C,D).

**FIGURE 5:**
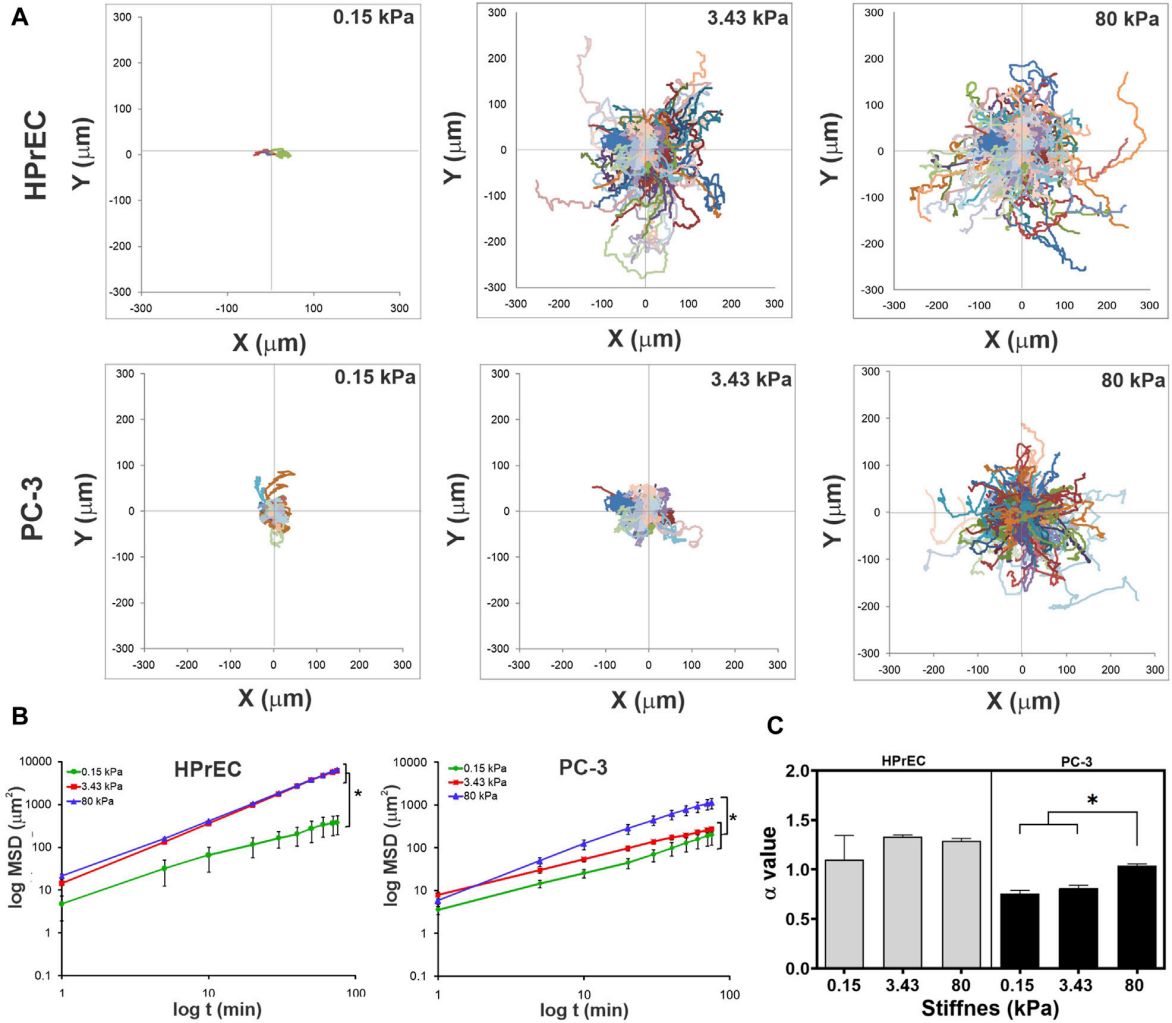
Modulation of migration persistence in non-tumoral and tumoral cells by substrate stiffness. **(A)** Trajectory plots of each migratory cell on PAA hydrogels after 150 min of recording time. **(B)** Log-log curves of MSD curves of HPrEC and PC-3 migratory cells on PAA hydrogels with different stiffness during the first 80 min of recording time. Data show mean ± SD, one-way ANOVA with Tukey’s multiple comparisons test. **(C)** Comparison of the α value (slope) of HPrEC and PC-3 migratory cells on PAA hydrogels with different stiffness; bars show mean ± SEM, one-way ANOVA with Tukey’s multiple comparisons test. Three independent experiments with, at least, two replicates per condition. *p < 0.05. HPrEC: 0.15 kPa, *n* = 3; 3.43 kPa, *n* = 157; 80 kPa, *n* = 160. PC-3: 0.15 kPa, *n* = 58; 3.43 kPa, *n* = 93; 80 kPa, *n* = 222.

Interestingly, trajectory plots of migrating cells confirmed that both HPrEC and PC-3 cells explore greater distances as stiffness increases. However, HPrEC cells explored a larger area on both intermediate and stiffer substrates compared to PC-3 cells, which explored a smaller area when cultured on the 3.43 kPa substrate (Figure 5A). Analysis of cell displacement using mean square displacement (MSD) demonstrated that HPrEC cells exhibited significantly larger displacements at 3.43 and 80 kPa, whereas for PC-3 cells, the difference was only observed at 80 kPa (Figure 5B). Notably, α values indicated that higher stiffness led to greater persistence of migration in PC-3 cells, whereas HPrEC cells did not show significant differences in α values across different substrate stiffnesses. However, the persistence values of HPrEC movement on intermediate and stiffer substrates were higher than those of PC-3 cells, suggesting that HPrEC cells maintained a straight, persistent movement (<1) unaffected by the stiffness, unlike PC-3 cells, which exhibited a more random movement (−1) on softer substrates (Figure 5C).

### 3.5 Stiffer substrates increase actin fiber formation and co-localization with NRP1 in tumoral PC-3 cells

**FIGURE 6:**
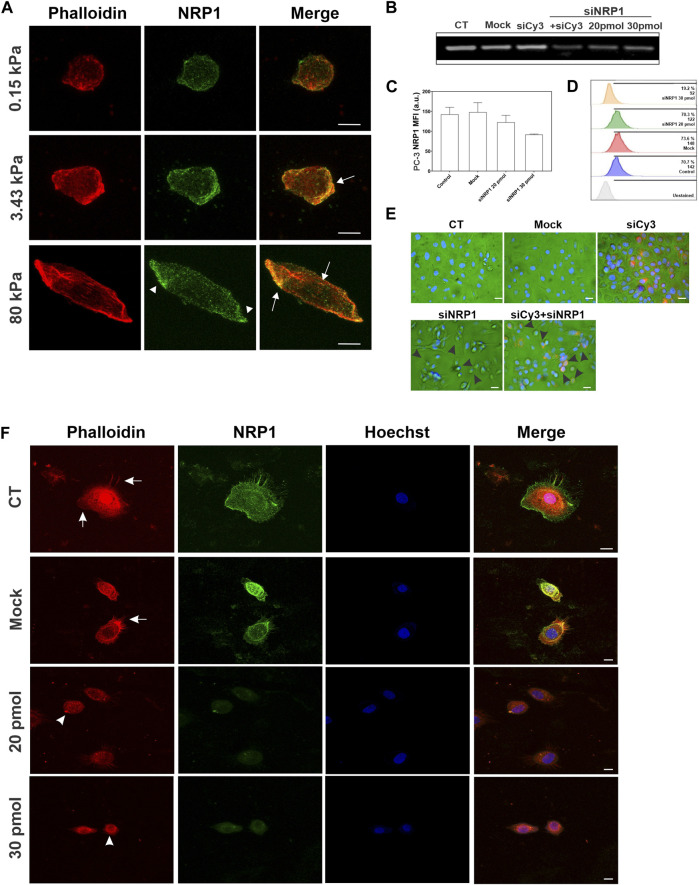
Actin and NRP1 distribution in tumoral PC-3 cells on different substrate stiffness, and the effect of NRP1 knockdown in actin distribution. **(A)** Representative confocal images of PC-3 cells cultured over coverslips and stained for actin (red) and NRP1 (green). Merged images show the co-localization of the two colors. **(B)** RT-PCR of PC-3 cells cultured over coverslips without transfection (CT), transfected without siRNA (Mock), transfected with a non-relevant siRNA coupled to Cy3 (siCy3), double transfected with siRNA-Cy3 and siRNA-NRP1 (siNRP1) 20 pmol, or only with siRNA-NRP1 at 20 and 30 pmol. **(C)** Plot showing the mean fluorescent intensity (MFI) of NRP1 measured by flow cytometry of control, mock, and silenced PC-3 cultured over coverslips. **(D)** Flow cytometry histograms showing the percentage of NRP1-positive PC-3 cells of control, mock, and silenced cell conditions. **(E)** Representative bright field and epifluorescence merge micrographs of transfected cells with siRNA-NRP1 or double transfected with siRNA-NRP1 and siRNA-Cy3 (red), nuclei were stained with Hoechst (blue). Arrowheads indicate transfected cells with a round morphology, as denoted by red staining. **(F)** Representative confocal images of PC-3 cells cultured over coverlips and stained for actin (red), NRP1 (green), and nuclei (blue), non-transfected (CT), transfected without siRNA (Mock), or with siRNA-NRP1 (20 pmol and 30 pmol). Arrows indicate actin in lamellipodia and filopodia, and arrowheads indicate actin aggregates in silenced cells. Scale bar = 10 µm.

The distribution and remodeling of the actin cytoskeleton play a crucial role in determining cell shape and migration. To characterize the distribution of actin and NRP1 in tumoral cells cultured on substrates with varying stiffness, we performed phalloidin staining and NRP1 immunostaining in PC-3 cells. We observed changes in polymerized actin as stiffness increased. On 0.15 kPa substrates, PC-3 cells exhibited few polymerized actin, mainly distributed as small dots and as subcortical actin. In contrast, in cells cultured on 3.43 and 80 kPa PAA hydrogels actin is distributed at the cells’ leading edges, in actin arcs and stress fibers, predominantly in cells on the stiffer substrate (Figure 6A). In the softest 0.15 kPa gels, NRP1 appeared as small dots distributed throughout the cells. However, on 3.43 and 80 kPa substrates, NRP1 localization was predominantly concentrated at the cells’ leading edge. Particularly noteworthy was the formation of larger NRP1 aggregates on the stiffer substrate (Figure 6A, arrowheads). Interestingly, NRP1 colocalized with actin at the polarized regions of the cells’ leading edges on 3.43 and 80 kPa substrates, as well as with the actin stress fibers under stiffer conditions (Figure 6A, arrows).

### 3.6 NRP1 knockdown impairs cell spreading and actin stress fibers formation in tumoral PC-3 cells

**FIGURE 7:**
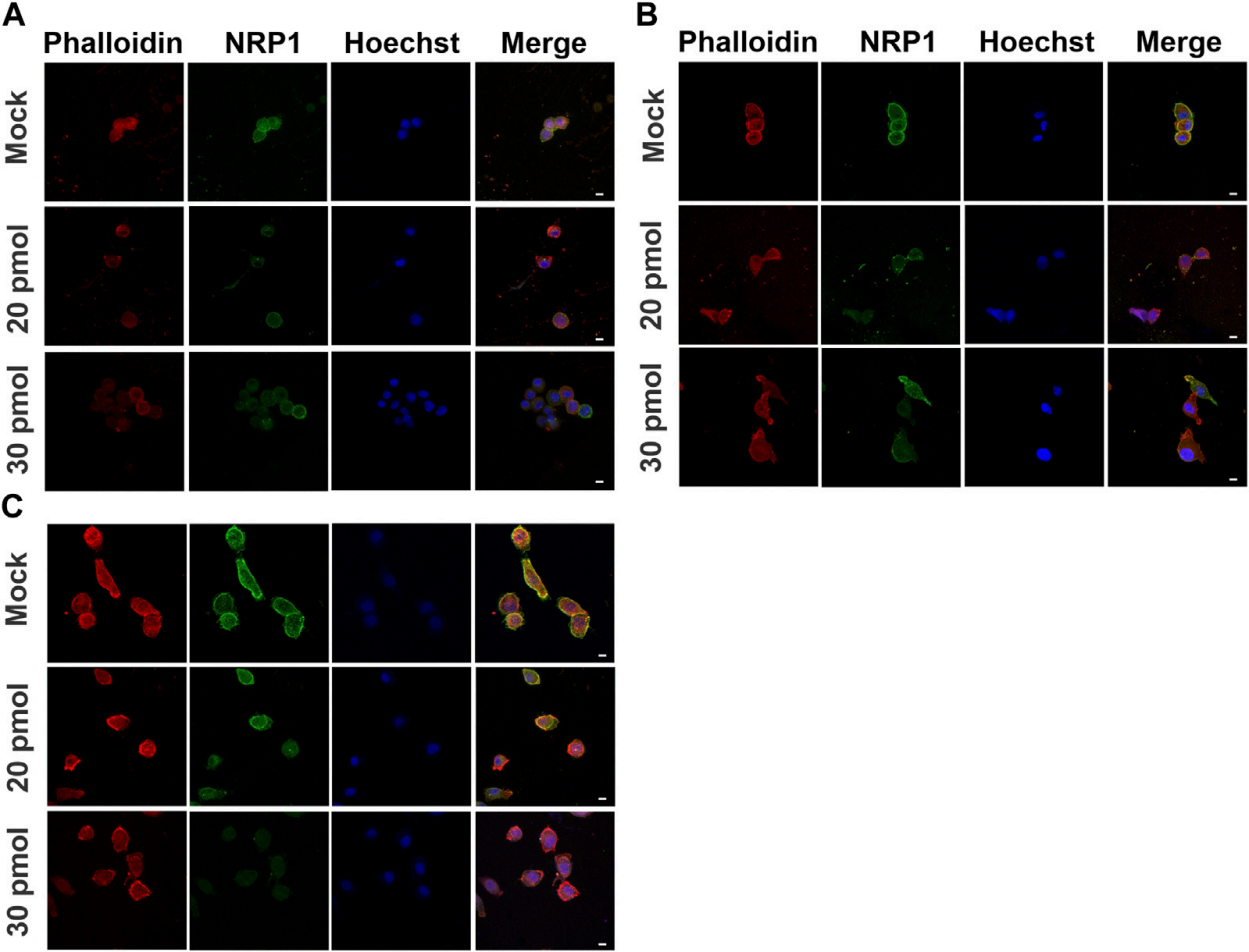
Actin distribution in NRP1 knockdown tumoral PC-3 cells on different substrate stiffness. Representative confocal images of PC-3 cells transfected without siRNA (mock), or with siRNA-NRP1 at 20 pmol or 30 pmol, and cultured for 48 h over **(A)** 0.15 kPa, **(B)** 3.43 kPa or **(C)** 80 kPa PAA hydrogels. Scale bar = 10 µm.

To investigate the role of NRP1 in cell morphology and cytoskeleton, we used siRNA targeting NRP1 to knock down its expression in tumoral PC-3 cells (Figures 6B–D). Upon transfection with siRNA-NRP1 and subsequent culturing on coverslips, we observed a decrease in cell spreading and an increase in rounded cell morphology (Figure 6E, arrowheads), as compared with cells with no relevant siRNA coupled to Cy3 or those lacking siRNA-NRP1. Evaluation of the impact of NRP1 knockdown on actin cytoskeleton distribution in cells cultured on coverslips revealed that non-silenced cells exhibited a spread morphology, characterized by lamellipodia and filopodia, along with strong cortical actin staining distributed in arcs and actin fibers (Figure 6F, arrows). In contrast, NRP1 knockdown cells displayed reduced spreading, assuming a rounded shape devoid of lamellipodia, and filopodia in many instances. Actin fibers were absent, with only scattered actin dots observed (Figure 6F, arrowheads). When we analyzed the effect of NRP1 knockdown on the cells cultured on the PAA hydrogels we observed similar trends, albeit less pronounced due to the inherently less spread morphology of cells compared to those on coverslips. On 80 kPa PAA hydrogels, silenced cells exhibited a more rounded morphology devoid of actin fibers, with diminished cortical actin and actin arcs. Some cells displayed only small aggregates of actin (Figure 7C). In contrast, effects were less evident in cells cultured on 3.43 and 0.15 kPa PAA hydrogels due to their less spread morphology and the lack of stress fibers, even in the non-silenced cells (Figures 7A,B).

### 3.7 NRP1 knockdown does not impair migration but modifies the protrusive mode of tumoral PC-3 migratory cells

**FIGURE 8:**
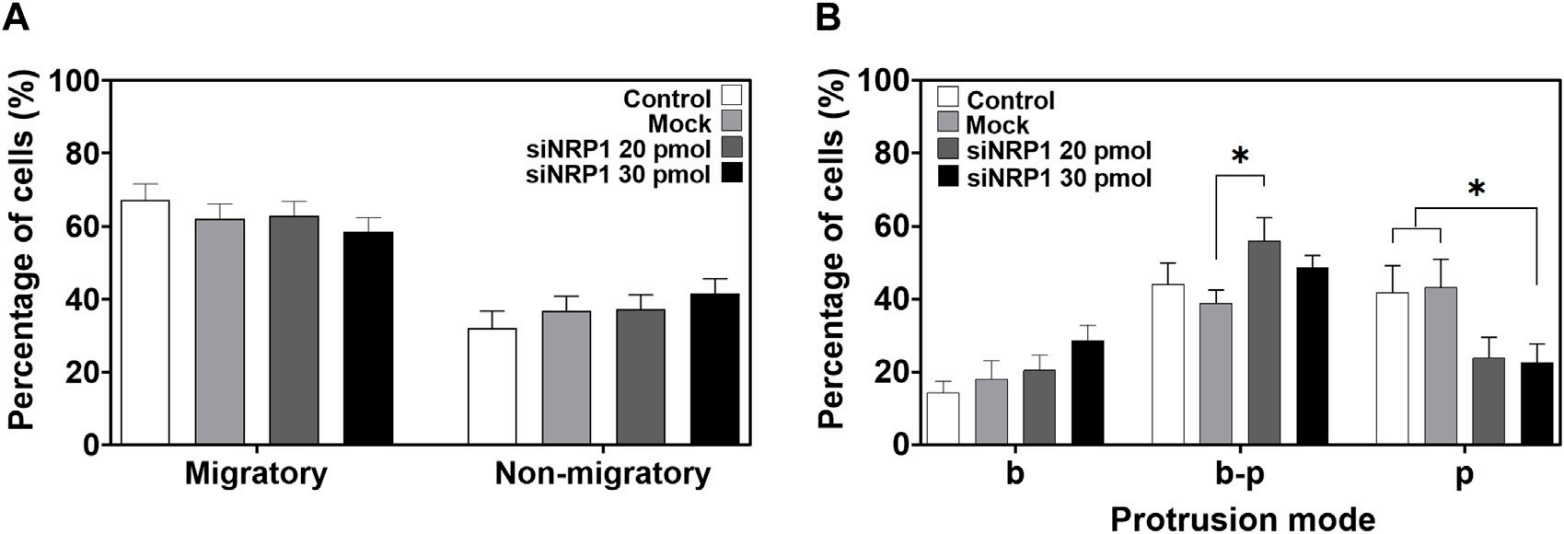
Effect of NRP1 knockdown in tumoral PC-3 cells migration and protrusion. **(A)** Plot showing the percentage of migratory and non-migratory PC-3 cells, non-transfected (Control), transfected without siRNAs (Mock), or with siRNA-NRP1 (siNRP1) 20 or 30 pmol. **(B)** Plot showing the percentage of cells with only blebs (b), bleb-pseudopodia (b–p), or only pseudopodia (p) protrusions in the non-transfected (Control), transfected without siRNAs (Mock), or with siRNA-NRP1 (siNRP1) 20 or 30 pmol cells. Data show mean ± SEM, Student’s t-test, *p < 0.05. CT, *n* = 8; Mock, *n* = 7; siRNA 20 pmol, *n* = 8; siRNA 30 pmol, *n* = 6 independent experiments.

To identify whether NRP1 plays a role in the migration of tumoral cells, we performed time-lapse imaging of silenced PC-3 cells cultured on 80 kPa gels. Surprisingly, NRP1 knockdown did not appear to hinder the number of migrating cells; the percentages of migratory *versus* non-migratory cells were similar between non-transfected and siRNA-NRP1 cultures (Figure 8A). Interestingly when we examined the modes of cell protrusion during migration. We found significant differences between silenced and control cells. siRNA-NRP1 led to a decrease in the number of cells exhibiting pseudopodia protrusions, while concurrently increasing the number of cells displaying a bleb-pseudopodia protrusive mode (Figure 8B, Supplementary videos SV-9, SV-10).

## 4 Discussion

The current study presents evidence of the differential impact of substrate stiffness on two tumoral and two non-tumoral cell lines, concerning the regulation of cell morphology, migration, and NRP1 expression, a protein associated with tumor progression. The stiffness of the extracellular microenvironment is currently considered a crucial factor in cell regulation. Numerous studies have demonstrated that the mechanical properties of the microenvironment play a significant role in cancer cells. Specifically, the extracellular stiffness regulates hallmarks of the cancerous process such as proliferation, survival, migration, and expression of tumoral markers (Northcutt et al., 2020). However, further investigations are needed to explore potential differences in stiffness responses regarding cell origin and malignancy. In this study, we assessed the effect of stiffness using substrates related to health-disease contexts, such as the normal cervical and prostate tissue (3.43 kPa) (Jiang et al., 2014; Piao et al., 2017), a prostate and cervical tumor (80 kPa) (Porsch et al., 2015; Cui et al., 2017; Rouvière et al., 2017), and a non-related stiffness, closer to nervous tissue stiffness (0.15 kPa) (Budday et al., 2020; Northcutt et al., 2020).

Our findings indicated that although the impact of substrate stiffness on cell morphology is similar for both tumorigenic and non-tumorigenic cell lines, there are differences in sensitivity to stiffness among cell lines. The impact of substrate stiffness on cell morphology has been extensively studied in a variety of normal and cancer cells (Lo et al., 2000; Flanagan et al., 2002; Engler et al., 2006; Solon et al., 2007). Alterations in cell morphology are linked to changes in cell cytoskeleton dynamics, facilitating the acquisition of malignant phenotypes (Carey et al., 2012; Pasqualato et al., 2013; Lyons et al., 2016). Previous studies have reported that stiffer substrates promote an increase in cell area and a more extended and elongated morphology in cancer cells (Schrader et al., 2011; Kristal-Muscal et al., 2013; Massalha and Weihs, 2017; Ansardamavandi et al., 2018). Prostate metastatic cancer cells DU145 and PC-3, as well as cervical cancer cells cultured on stiffer substrates (2.92 MPa and 20 kPa, respectively), also exhibit responses to stiffness, characterized by increased cell surface area and the presence of more pseudopodia, particularly in cervical cells (Missirlis, 2014; Prauzner-Bechcicki et al., 2015; Piao et al., 2017; Jin et al., 2022). Similarly, our observations reveal comparable morphological changes; however, a more pronounced increase in area and decrease in roundness was observed at 3.43 kPa in tumoral PC-3 cells compared to tumoral HeLa cells. This result could be related to the bone-metastatic origin of PC-3 cells compared to non-metastatic HeLa cells, suggesting a potential correlation with the cells’ invasive capacity into healthy tissues that are usually softer than the prostatic tumor environment, coupled with greater cell deformability, thereby facilitating motility and invasiveness (Pasqualato et al., 2013). Likewise, when comparing two non-tumorigenic cell lines, the results indicate a more sensitive response of HPrEC compared to HEK293, probably related to the stiffness of their respective tissue origins.

On the other hand, we report, for the first time, the impact of stiffness on NRP1 expression in both non-tumoral and tumoral cells. Despite the evidence concerning the role of NRP1 in cancer progression and poor prognosis, it has not been reported whether extracellular stiffness correlates with NRP1 expression in cancer cells. Previously, in dorsal root ganglion neurons, we observed a direct relationship between stiffness and the upregulation of NRP1 mRNA expression, which in turn modulates the response to the chemotropic protein semaphorin 3A (Vela-Alcántara et al., 2022). The findings of this study underscore the relevance of stiffness in regulating NRP1 expression. Our results show that NRP1 mRNA expression increases as the substrate becomes stiffer in non-tumoral HPrEC and HEK293 cells. However, HeLa and PC-3 cells exhibit no statistically significant differences in mRNA levels across the different substrate stiffness. Notably, PC-3 cells display an earlier increase in the NRP1 expression at normal prostate tissue stiffness. Previous studies have reported NRP1 overexpression in tumoral prostate cancer cell lines such as LNCaP, DU145, and PC-3, compared with benign prostatic epithelial cell lines cultured on conventional tissue culture plates (Latil et al., 2000; Tse et al., 2017). However, conclusive reports regarding NRP1 expression in HeLa and HEK293 cell lines are lacking, although both cell lines have been reported to exhibit low NRP1 expression (Barman et al., 2019). There are no previous reports on NRP1 expression in HPrEC cells. Remarkably, during cancer progression, a significant increase in stiffness is driven by the accumulation and reorganization of extracellular matrix components and fibrosis. Moreover, fibrotic tissue can be a tumor-supportive microenvironment for metastatic cells Cox et al., 2021). NRP1 is associated with increased matrix stiffness by promoting fibronectin reorganization and collagen secretion (Cao et al., 2010; Yaqoob et al., 2012), mediated by its role as a coreceptor for β1 integrins (Valdembri et al., 2009) and TGF-β1 (Glinka et al., 2011); the last one a relevant growth factor for myofibroblasts recruitment and tumor fibrotic response (Cao et al., 2010; Kojima et al., 2010; Papageorgis and Stylianopoulos, 2015). Additionally, substrate stiffness is known to regulate the expression of genes related to cancer progression, including the growth factor VEGF_165_, one of the ligands of NRP1 (Sack et al., 2016), and some integrins such as α5β4 (Schrader et al., 2011), which, when overexpressed and bound to NRP1, promote ECM remodeling, induce tumor growth, and contribute to desmoplasia and stiffening in tumors (Yaqoob et al., 2012). Our findings suggest that NRP1 regulation by stiffness creates a feedback loop promoting stiffer substrates and enhancing cell malignancy. It is noteworthy that NRP1 mRNA expression significantly increased in non-tumoral prostate epithelial cells HPrEC when subjected to stiffness related to a prostate tumor microenvironment (80 kPa). Conversely, the tumoral metastatic cell line PC-3 exhibits NRP1 mRNA overexpression at a stiffness resembling normal tissue (3.43 kPa). While these results suggest differential mechanosensitive and regulatory mechanisms of NRP1 induction by stiffness in non-tumoral and tumoral cells, further investigation comparing the response of normal and tumoral cells from the same individual samples is important to exclude factors such as genetic profiles or other non-tumoral related factors. Besides, our results suggest that the impact of stiffness on NRP1 expression is predominantly due to *de novo* protein induction rather than an increase in expression levels within already NRP1-positive cells. Fluorescence intensity analysis across different stiffnesses showed no significant changes except in PC-3 cells, indicating that cells do not significantly modify the number of receptors on the cell membrane. Previous research has highlighted the importance of *de novo* expression of genes related to cytoskeleton components and focal adhesions, such as actin and vinculin, during migration and that this regulation is modulated by activation of mechanotransducer YAP/TAZ through the ROCK signaling pathway (Mason et al., 2019). Interestingly, a recent study has shown NRP1 induction via mechanical compression, unveiling for the first time its interaction with YAP. Stress induced by mechanical compression disrupts NRP1/YAP interaction, thereby hindering hypertrophic scar development by inhibiting angiogenesis (Li et al., 2023). Investigating whether stiffness-induced NRP1 expression involves mechanotransducer pathways, including proteins like YAP and the activation of the Rho and ROCK signaling pathways, which also facilitate NRP1 ligand activation and thus promote cancer cell malignancy, could provide valuable insights.

Additionally, our results showed that tumoral cells experience an increase in actin cytoskeleton fiber formation and polarized actin distribution with increasing stiffness, influencing NRP1 distribution and its co-localization with actin. A stiffer tumor microenvironment enhances cell migration through the regulation of integrins, the maturation of focal adhesion, and downstream cascades that induce cytoskeleton remodeling (Gkretsi and Stylianopoulos, 2018). A stiffness increase could promote cell polarization and retrograde actin flow, as observed in MDA-MB-231 cells (Isomursu et al., 2022). Although NRP1 and actin co-localization have been reported in DRG neurons growth cones after exposure to semaphorin 3A (Fournier et al., 2000), and in endothelial cells exposed to semaphorin 3C (Salikhova et al., 2008), this study presents the first evidence of such co-localization in tumoral cells across different substrate stiffness. We also observed that an increase in substrate stiffness significantly enhances migratory behavior in PC-3 cells, with a more than fourfold increase at 80 kPa substrate, compared to cells on softer conditions, while HPrEC cells exhibited a higher percentage increase of migratory cells under the intermediate stiffness condition (3.43 kPa). Furthermore, the cells modify their membrane protrusions depending on substrate stiffness. PC-3 cells transit from bleb-pseudopodia at 3.43 kPa to predominantly pseudopodia at 80 kPa, while HPrEC cells exhibit no change between these conditions. Tumor cells exhibit versatility in motility, transitions between mesenchymal, characterized by elongated shapes and pseudopodia, and ameboid motility, marked by rounded morphology and bleb-based migration (Friedl and Wolf, 2010). Bleb protrusion is related to increased cortical tension and decreased cell adhesion, while pseudopodia formation is related to actin polymerization and adhesiveness (Bergert et al., 2015). Our findings revealed that the NRP1-silenced PC-3 cells have a significant decrease in the number of cells with pseudopodia protrusions; moreover, the cells lose lamellipodia, decrease their filopodia, and increase the bleb-pseudopodia protrusion mode, suggesting NRP1’s role in pseudopodia regulation and potentially actin cytoskeleton regulation at cells protrusions. Earlier research has linked NRP1 to actin remodeling in response to fibronectin substrate exposure, with NRP1 silencing impairing endothelial cell spreading, filopodia extension, and migration (Raimondi et al., 2014). Furthermore, it has been reported that endothelial cell sprouting and filopodia formation in response to ECM is mediated by NRP1 activation of CDC42, promoting actin remodeling and filopodia formation (Fantin et al., 2015).

The observed changes in protrusion modes in PC-3, driven by stiffness, suggest a higher mechanical adaptation and cell plasticity potentially mediated by NRP1 overexpression that enhances migration. This underscores the tumoral cell’s ability to respond to physical changes in the microenvironment compared to non-tumoral cells. Mean square displacement (MSD) analysis further shows that stiffness also increases the area explored by cells, with HPrEC cells exploring a larger area than PC-3 cells, even at intermediate stiffness. However, only PC-3 cells show increased directional displacement on stiffer substrates. This directional persistence analysis is suitable for measuring the migration of cells, whether migration is guided by chemotactic gradients or other external cues, and therefore reflects the directionality of the cell movement *versus* random movement (Gorelik and Gautreau, 2014). Our results align with a prior study comparing different prostate cancer cell lines, which shows that PC-3 cells exhibit increased migration persistence as stiffness increases. However, in that report, the migration assays were performed on cell monolayers (Molter et al., 2022), which are more relevant to collective migration assays, compared with the current study where the analysis was conducted on individual cells and at higher stiffness. These results suggest that PC-3 cells respond to an increase in substrate stiffness through a durotaxis-like response mechanism (Petrie et al., 2009), which involves cell adhesion mediated by cytoskeleton mechanics through retrograde actin flow and activation of focal adhesion complexes to generate the necessary traction force for cell migration on stiff substrates. In this process, YAP/TAZ mechanotransducers play a crucial role in modulating intracellular tension and facilitating the focal adhesion polarization and consolidation at the cell’s leading edge, thereby generating the necessary tension to maintain a persistent motility (Mason et al., 2019).

## 5 Conclusion

In conclusion, we add evidence to the impact of stiffness related to tumoral tissue on cell morphology, actin distribution, and migration, and for the first time, in NRP1 expression, a malignancy-related protein, and its co-localization with actin. Furthermore, the differential stiffness responses between non-tumoral and various tumoral cell lines add evidence about the importance of investigating the mechanical response heterogeneity in cancer cells. The findings presented here also support the role of NRP1 as a malignancy biomarker and highlight the potential of developing new strategies for the design and use of mechanical tumor microenvironment regulators as a potential strategy to modulate the expression of proteins involved in cancer progression.

## Data Availability

The original contributions presented in the study are included in the article/Supplementary Material, further inquiries can be directed to the corresponding author.
