# Supplementary material for: Differential modulation of cell morphology, migration, and Neuropilin-1 expression in cancer and non-cancer cell lines by substrate stiffness
Source: Front Cell Dev Biol. 2024 Jun 5;12:1352233. doi: 10.3389/fcell.2024.1352233 (PMC11188430; doi:10.3389/fcell.2024.1352233)
Supplement: Supplementary file 5 [file DataSheet1.PDF]

Supplementary Material

Differential modulation of cell morphology, migration, and Neuropilin-1 expression in cancer and non-cancer cell lines by substrate stiffness

Ana Monserrat Vela-Alcántara[1,2], Juan Santiago-García[3], Madeleine Barragan-Palacios[2, 4], Aylin León-Chacón[2], Marilú Domínguez-Pantoja[5], Enrique Juárez-Aguilar[2], Elisa Tamariz[2]*

***Correspondence:**

Elisa Tamariz
etamariz@uv.mx

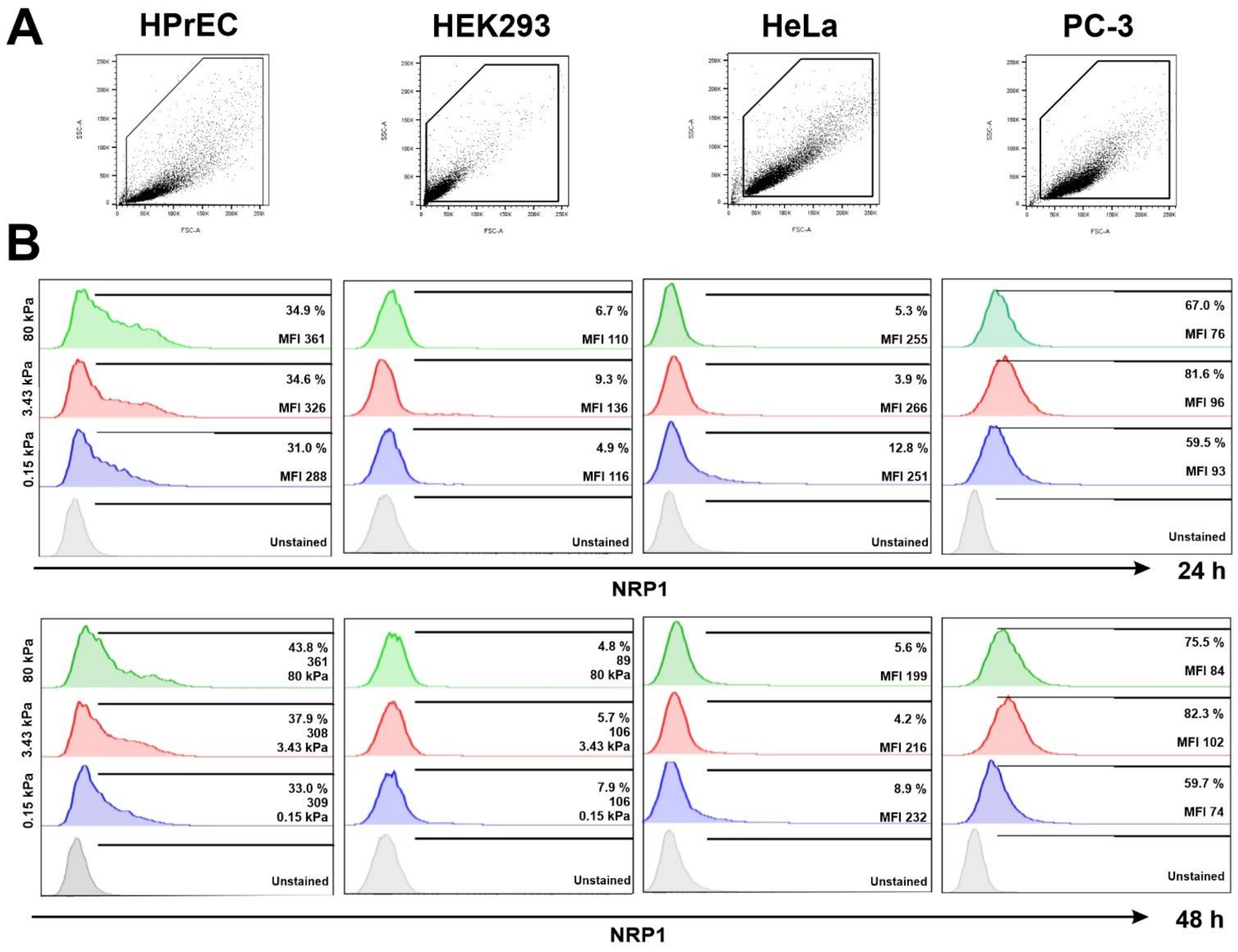

Supplementary Figure 1. (A) Representative flow cytometry dot plots showing HPrEC, HEK293, HeLa, and PC-3 cell populations. (B) Representative histograms showing the percentage of NRP1 positive cells and NRP1 mean fluorescent intensity (MFI) of HPrEC, HEK293, HeLa, and PC-3 cells cultured for 24 h and 48 h on PAA hydrogels.

Supplementary Videos. Representative videos of HPrEC and PC3 cells on PAA hydrogels
SV1: HPrEC cells on 0.15 kPa PAA hydrogel. **SV2**: HPrEC cells on 3.43kPa PAA hydrogel. **SV3**: HPrEC cells on 80kPa PAA hydrogel. **SV4**: PC3 on 0.15 kPa PAA hydrogel. **SV5**: PC3 on 3.43kPa PAA hydrogel. **SV6**: PC3 on 80 kPa PAA hydrogel. **SV7**: PC3 on 80kPa without transfection. SV8: PC3 on 80kPa transfected without siRNAs. **SV 9:** PC3 on 80kPa transfected with 20pM of siRNA-NRP1. **SV 10:** PC3 on 80kPa transfected with 30pM of siRNA-NRP1.
